# Rapid measurement of direct oral anticoagulants for clinical decision-making

**DOI:** 10.1016/j.rpth.2026.106997

**Published:** 2026-10-01

**Authors:** Elizabeth Ronchetto, Jean Connors, Gabriel V. Fontaine, Kamran Hajiyev, Steve Kitchen, David Z. Rose, Scott Kaatz

**Affiliations:** 1 Department of Internal Medicine, Henry Ford Hospital, Detroit, Michigan, USA; 2 Division of Hematology, Dana-Farber Cancer Institute, Boston, Massachusetts, USA; 3 Department of Pharmacy, Intermountain Medical Center, Intermountain Health, Salt Lake City, Utah, USA; 4 Department of Neuroradiology, Klinikum Stuttgart, Stuttgart, Baden-Württemberg, Germany; 5 UK NEQAS Blood Coagulation, World Federation of Hemophilia IEQAS, Sheffield, UK; 6 Department of Neurology, University of South Florida Morsani College of Medicine, Tampa General Hospital, Tampa, Florida, USA; 7 Division of Hospital Medicine, Henry Ford Hospital, Detroit, Michigan, USA

**Keywords:** blood coagulation tests, clinical decision-making, direct oral anticoagulants, factor Xa inhibitors, ischemic stroke, point-of-care testing

## Abstract

**Background:**

Direct oral anticoagulants (DOACs) are widely used for the prevention and treatment of thromboembolic disease and have streamlined long-term anticoagulation management due to fixed dosing. However, in time-sensitive clinical scenarios such as acute ischemic stroke, major bleeding, and perioperative care, uncertainty regarding anticoagulant effect can complicate decision-making, such as treatment with thrombolytic and/or reversal agents.

**Objectives:**

This review summarizes the limitations of existing measurement strategies, outlines key clinical scenarios where rapid assessment may inform care, and evaluates the potential role of point-of-care testing in improving safety and decision-making in hyperacute anticoagulation management.

**Methods:**

We assessed literature evaluating laboratory and point-of-care methods for assessing DOAC activity, with emphasis on their application in clinical settings, including acute ischemic stroke, major bleeding, and perioperative management.

**Results:**

Current laboratory approaches to assess DOAC activity, including routine coagulation tests and DOAC-specific assays, are limited by variable sensitivity, limited availability, delayed turnaround times, and lack of validated therapeutic thresholds to guide management. As a result, clinicians must make decisions without reliable, timely information on anticoagulant status. Emerging point-of-care technologies offer the potential to address this gap by providing rapid, clinically actionable insights into DOAC presence and effect.

**Conclusion:**

Rapid measurement of DOAC activity has the potential to improve clinical decision-making in time-sensitive scenarios involving patients receiving DOAC therapy. Future efforts should focus on integrating these approaches into clinical practice and evaluating their impact on patient-centered outcomes.

## Essentials

• Uncertainty regarding direct oral anticoagulant (DOAC) activity can complicate urgent clinical decision-making.
• Rapid DOAC assessment may inform care in stroke, major bleeding, and perioperative settings.
• Current laboratory methods to measure DOAC activity have important limitations.
• Emerging point-of-care tests may improve timely clinical decision-making.

## 1 Introduction

Direct oral anticoagulants (DOACs) are the predominant form of oral anticoagulation therapy in clinical practice in many countries. DOACs include the oral factor Xa inhibitors apixaban, rivaroxaban, and edoxaban and the oral direct thrombin inhibitor dabigatran [1]. Prescribing of DOACs has increased year over year since their introduction in the early 2010s and is expected to continue to rise as the prevalence of atrial fibrillation (AF) increases with the aging population [2], [3], [4]. Compared with vitamin K antagonists and low-molecular-weight heparin, DOACs offer a more favorable safety profile with lower bleeding risk [5], [6]. Although routine clinical monitoring for bleeding, thrombosis, renal function, and medication adherence is important, DOACs do not require routine laboratory measurement of anticoagulant effect because of their predictable pharmacokinetics. Nonetheless, in certain clinical scenarios, rapid measurement of anticoagulant activity from DOAC use could inform care. Routine coagulation assays may be affected by DOAC exposure; however, their sensitivity and magnitude of response vary among reagents and laboratories, limiting their reliability for quantifying the anticoagulation effect. Additionally, current laboratory techniques, such as DOAC-sensitive anti-Xa assays, are not widely or rapidly available in many centers. In several clinical scenarios, including hyperacute stroke care, bleeding events, and upcoming surgical or procedural interventions, whether emergent or routine, knowledge of anticoagulant effect may guide decision-making by answering a key question: Is this patient anticoagulated, and if so, to what degree?

## 2 Doac Pharmacokinetic Reference Ranges

Therapeutic ranges for DOAC concentrations have not been established. However, on-therapy peak and trough concentrations have been identified, as shown in Table 1. These peak and trough levels were derived from pharmacokinetic modeling of phase II and III clinical trials or plasma levels of drugs in small cohorts of healthy volunteers [11], [12]. Participant demographic characteristics, including race and ethnicity, are not presented in Table 1, which represents a limitation of the summarized data. These reported concentrations represent pharmacokinetic reference values rather than validated therapeutic targets. In practice, clinicians extrapolate these pharmacokinetic data to interpret measured DOAC effect, but this approach is limited by the lack of clinical outcome data. As a result, no universal recommendations exist for dose adjustments or treatment decisions based on DOAC concentrations. Some studies have associated higher DOAC concentrations with bleeding events and lower concentrations with thrombotic events, but no large prospective studies have established a correlation between thrombosis or bleeding and DOAC concentrations as measured by anti-Xa [12], [13].

**Table 1:** Expected on-therapy peak and trough direct oral anticoagulant concentrations by indication and dose.

DOAC	Indication	Dose	Peak (ng/mL)	Trough (ng/mL)
Apixaban	Atrial fibrillation	5 mg twice a day Gosselin et al. [7]	171 (91-321)^a^	103 (41-230)^a^
		2.5 mg twice a day Cirincione et al. [8]	123 (69-221)^a^	79.2 (34-221)^a^
	VTE/PE treatment	5 mg twice a day Gosselin et al. [7]	132 (59-302)^a^	63 (22-177)^a^
Edoxaban	Atrial fibrillation	60 mg daily Gosselin et al. [7] Verhamme et al. [9]	170 (125-245)^b^	36 (19-62)^c^
	VTE/PE treatment	60 mg daily Gosselin et al. [7] Verhamme et al. [9]	234 (149-317)^c^	19 (10-39)^c^
Rivaroxaban	Atrial fibrillation	20 mg daily Gosselin et al. [7] Mueck et al. [10]	249 (184-343)^d^	44 (12-137)^d^
	VTE/PE treatment	20 mg daily Gosselin et al. [7] Mueck et al. [10]	270 (189-419)^d^	26 (6-87)^d^
Dabigatran	Atrial fibrillation	150 mg twice a day Gosselin et al. [7]	175 (117-275)^e^	91 (61-143)^e^
	VTE/PE treatment	150 mg twice a day Gosselin et al. [7]	175 (117-275)^e^	60 (39-95)^e^

PE, pulmonary embolism; VTE, venous thromboembolism.

a Median (5th-95th percentile).

b Median (1.5 × IQR).

c Median (IQR).

d Mean (5th-95th percentile).

e Mean (25th-75th percentile).

The optimal DOAC concentration cutoff for minimal levels to guide clinical decision-making is also not outcome-validated, though thresholds of 30 and 50 ng/mL are most frequently used as the lower limit of detection [14], [15], [16], [17]. These cutoffs of 30 and 50 ng/mL are derived from expert and consensus opinions, using results from pharmacokinetic data and post hoc phase III clinical trial analyses. Notably, the International Society on Thrombosis and Haemostasis endorses the 30 to 50 ng/mL range when recommending reversal agent use [14]. For urgent surgeries with hemorrhagic risk, levels below 30 ng/mL are generally acceptable to proceed [15], [16]. The American Society of Regional Anesthesia and Pain Medicine guidelines state residual DOAC levels of less than 30 ng/mL are generally acceptable prior to neuraxial block procedures [17].

Several studies have evaluated DOAC concentrations in clinical settings, aiming to compare real-world laboratory values with those observed in clinical trials and highlight inter and intrapatient variability in DOAC concentrations [18], [19]. One multicenter study evaluated peak and trough anti-Xa levels in 99 patients receiving apixaban and 71 receiving rivaroxaban, revealing interpatient variability with coefficient of variation values ranging from 21% to 42% at peak and 29% to 68% at trough for apixaban and 32% to 52% at peak and 30% to 103% at trough for rivaroxaban [19]. This variability may reflect differences in pharmacokinetics and dosing regimens between agents, including twice-daily DOACs (apixaban and dabigatran) and the once-daily DOAC rivaroxaban. However, in randomized trials evaluating DOACs vs vitamin K antagonists for stroke and systemic emboli in AF, apixaban and dabigatran were each shown to be superior to warfarin, driven primarily by a decrease in hemorrhagic stroke, whereas once-daily DOACs (rivaroxaban and edoxaban) were shown to be noninferior [20], [21], [22], [23].

At lower drug concentrations, interassay variability is also a concern, although the clinical relevance remains uncertain [24]. Anti-Xa levels for apixaban and rivaroxaban measured across 3 commonly used instrument-reagent combinations were generally well correlated; however, a statistically significant variability emerged at the 30 ng/mL threshold. Ten of 129 samples (7.8%) crossed the 30 ng/mL cutoff, depending on the assay used, with the largest difference in samples being 12 ng/mL. Importantly, none of these discrepant samples crossed the 50 ng/mL threshold [25]. However, evaluation of only 3 assays may not fully capture the interassay variability that could emerge across a broad range of reagent-instrument combinations.

## 3 Clinical Conundrums

### 3.1 Acute ischemic stroke care

#### 3.1.1 Is the degree of DOAC anticoagulation low enough to administer intravenous thrombolytics or to perform intracranial interventions in acute ischemic stroke (AIS)?

For patients with nonvalvular AF, DOACs reduce the risk of stroke or systemic embolism compared with vitamin K antagonists, which had been the mainstay of preventive therapy for decades [20], [21], [22], [23]. Nevertheless, stroke or systemic embolism occurred in 4 landmark AF-stroke trials at an annual rate of 1.1% (dabigatran), 1.7% (rivaroxaban), 1.3% (apixaban), and 1.2% (edoxaban) [20], [21], [22], [23]. Colloquially referred to as “DOAC failure,” observational cohorts have described a higher incidence of breakthrough AIS in real-world settings [26], [27], [28], [29].

In the hyperacute phase of ischemic stroke, intravenous thrombolysis (IVT), with alteplase or tenecteplase, is the standard of care, barring contraindications and time-window restrictions. IVT improves long-term functional outcomes and decreases mortality risk [30], [31]. Mechanical thrombectomy has likewise demonstrated clinical benefits for patients with AIS with large-vessel occlusion [32]. However, anticoagulant use introduces complexity into IVT and mechanical thrombectomy decisions. The 2019 American Heart Association and American Stroke Association (AHA/ASA) guidelines advise against IVT in patients with AIS who have taken a DOAC within the preceding 48 hours, unless appropriate coagulation testing is normal [33]. In the updated 2026 AHA/ASA ischemic stroke guidelines, recent DOAC exposure (within 48 hours) is a relative contraindication to IVT. This updated recommendation reflects the fact that patients treated with DOACs were excluded from the large trials supporting use of IVT, leaving uncertainty about bleeding risk in this population. However, accumulating observational data suggest IVT may be safe in select patients with recent DOAC exposure [34], [35]. The 2026 AHA/ASA ischemic stroke guidelines recognize this as an important area for further research [36].

Given the lack of randomized studies addressing stroke management in patients treated with DOACs, both guidelines and clinical practice are largely informed by retrospective studies and expert consensus. A large, multicenter, retrospective observational study comparing AIS outcomes following alteplase administration in patients receiving DOAC therapy vs those not receiving DOACs found lower mortality in the DOAC group [34]. Notably, the criteria used to determine eligibility for thrombolysis among patients treated with DOACs were not clearly identified, despite existing stroke guidelines. These findings and other similar retrospective reviews [35] have led some experts to suggest that recent DOAC use should not alone constitute an absolute contraindication to IVT therapy [34]. Still, in clinical practice, recent DOAC exposure frequently remains a barrier to receiving IVT for AIS in otherwise eligible patients. DOAC use is generally not considered a contraindication for mechanical thrombectomy in otherwise eligible patients, though uncertainty regarding anticoagulant activity may still influence procedural decision-making, and DOAC-associated intracranial hemorrhage or transformation of AIS in the mechanical thrombectomy population remains unknown [37].

Coagulation laboratory tests may offer some guidance regarding thrombolytic therapy for DOAC users [36]. Ideally, such testing would accurately reflect the anticoagulant effect and be readily and rapidly available. Timely determination of anticoagulation status may aid clinician decision-making in patients with AIS when DOAC exposure is uncertain. If a patient’s DOAC use is unclear at the time of considering IVT, whether due to preexisting or new cognitive deficits, expressive or receptive aphasia, or other communication limitations related to stroke symptoms, or the absence of reliable collateral information from caregivers, then rapid assessment of DOAC effect may vitally aid hyperacute clinical management.

Patients with high-risk pulmonary embolism requiring systemic thrombolysis may represent another clinical scenario in which rapid assessment of anticoagulant activity may influence treatment decisions. Although evidence is more limited than in AIS, knowledge of clinically relevant DOAC activity may help balance thrombotic and bleeding risks when thrombolysis is being considered.

### 3.2 Bleeding

#### Is the degree of DOAC anticoagulation low enough to avoid reversal?

Is the degree of DOAC anticoagulation high enough to warrant reversal? Although DOACs in general carry a similar-to-lower bleeding risk than vitamin K antagonists, bleeding remains the most common and concerning complication associated with DOAC use [21], [22], [23]. In the United States, nearly 200,000 emergency department visits occurred for DOAC-related bleeding in 2020, higher than in prior years [38]. Hence, as DOAC use continues to increase, DOAC-related bleeding events will also increase [2], [3], [4].

Major bleeding is defined by the International Society on Thrombosis and Haemostasis as fatal bleeding, overt bleeding resulting in a decrease in hemoglobin of at least 2 g/dL or requiring transfusion of at least 2 units of packed red blood cells, or symptomatic bleeding occurring in critical areas such as intracranial or retroperitoneal sites [39]. Clinical trial data in patients with AF demonstrate rates of major bleeding ranging from 2.13% to 3.6% per year in DOAC-treated groups, with intracranial hemorrhage rates of 0.33% to 0.85% per year [21], [22], [23]. Gastrointestinal bleeding is the most common source of both major bleeding noted in clinical trials and the most common reason for DOAC-related bleeding emergency department visits [21], [22], [23], [38]. Studies have found similar rates of bleeding in clinical settings compared with clinical trial-reported bleeding rates [40].

When bleeding is severe or life threatening, rapid treatment decisions are required. In such cases, interventions such as prothrombin complex concentrate or specific DOAC reversal agents, idarucizumab for dabigatran or andexanet alfa for factor Xa inhibitors, can rapidly counteract DOAC effects. Markedly elevated DOAC concentrations may also have implications for the effectiveness of reversal. Incomplete reversal with idarucizumab has been reported with significantly elevated dabigatran concentrations (>800 ng/mL) [41]. These reversal therapies, however, carry risks, most notably thrombosis, including AIS, myocardial infarction, deep vein thrombosis, and pulmonary embolism. The overt cost of reversal agents adds another important consideration to management decisions. Furthermore, in scenarios of life-threatening bleeding, especially intracranial hemorrhage or blood-loss anemia resulting in hemorrhagic shock, patients may be confused, obtunded, or even comatose and unable to communicate their medication history. Rapid results based on individual patient testing of whether a clinically relevant DOAC effect is present would support more informed and timely clinical decision-making and potentially avoid excess thrombosis risk.

Notably, the reversal agent idarucizumab, designed to neutralize the DOAC dabigatran, is a selective humanized monoclonal antibody and therefore does not carry such a thrombosis risk. Indeed, some centers have reported use of idarucizumab pre-IVT in patients with AIS on dabigatran; one case series found a stroke scale improvement between 2 and 5 points with this strategy, resulting in penumbral salvage of ischemic brain tissue on neuroimaging ranging from 63.4% to 97.8% [42].

It should be noted that a specific reversal agent for the oral factor Xa inhibitors rivaroxaban, apixaban, and andexanet alfa has been withdrawn from the market in the United States, with withdrawal in other countries expected soon.

### 3.3 Emergent/urgent surgery or procedure

#### 3.3.1 Is the degree of DOAC anticoagulation low enough to avoid reversal?

Is the degree of DOAC anticoagulation high enough to warrant reversal? Regardless of recent DOAC exposure or clinically relevant DOAC concentrations, patients may require emergent operative or procedural intervention. In these cases, DOAC reversal may be considered prior to the intervention to mitigate peri-procedural bleeding risk. Expectedly, in many scenarios, recent DOAC exposure may be unknown, whether due to baseline cognitive impairment, critical illness impacting ability to communicate, or questionable adherence. As a result, clinicians may be required to make perioperative decisions with incomplete information. Rapid knowledge of clinically relevant DOAC effects may aid clinical decision-making around preoperative reversal. Furthermore, even if DOAC reversal is not being considered preoperatively due to the need for immediate intervention, availability, or side effects, this information may be helpful if bleeding were to become an issue intraoperatively or in the perioperative period. Knowledge of clinically relevant DOAC activity may also facilitate multidisciplinary communication and procedural planning by anticipating bleeding risk and informing contingency planning should reversal become necessary.

Measurement may also identify unexpectedly elevated DOAC concentrations despite reported interruption of therapy. Such findings should not only inform perioperative management but should prompt reassessment of factors contributing to excessive drug exposure, including renal or hepatic dysfunction, heart failure, drug–drug interactions, or other causes of impaired clearance. Recognition of markedly elevated concentrations may influence subsequent anticoagulation management.

#### 3.3.2 Is the degree of DOAC anticoagulation low enough to proceed with routine or elective intervention?

Patients are instructed to stop taking their DOAC for a specified number of days prior to an elective surgery or procedure, then resume it a specified number of days afterward. The duration is based on the bleeding risk of the intervention and patient-specific factors, including their risk of thrombosis while holding anticoagulation. The Perioperative Anticoagulation Use for Surgery Evaluation (PAUSE) trial addressed perioperative management of apixaban, rivaroxaban, and dabigatran in patients with AF undergoing elective interventions. DOAC-specific anti-Xa assays were used to determine residual anticoagulant levels prior to the procedure. Overall, 98.8% of the total 2541 patients in the study had residual anticoagulant levels less than 50 ng/mL across the apixaban, dabigatran, and rivaroxaban groups. However, in the high-bleeding-risk procedure group, residual levels of 30 to 49.9 ng/mL were noted to be 4.8% in the apixaban cohort and 14% in the rivaroxaban cohort [43]. More recently, a cohort study including over 250 surgical patients instructed to follow an interruption strategy prior to their surgery identified 7.6% of patients with residual DOAC levels at or above 30 ng/mL [44].

DOAC anti-Xa levels are not routinely checked prior to scheduled interventions. Consistent with current American College of Chest Physicians perioperative guidelines, routine measurement of anticoagulant activity is not recommended [45]. As noted in the PAUSE trial, the simple interruption strategy used was associated with low rates of major bleeding and arterial thromboembolism. Despite interruption strategies, a small subset of patients had greater than 30 ng/mL of residual drug despite holding their DOAC [43], [44]. Furthermore, adherence to stopping a DOAC as instructed prior to an intervention may be in question for some patients. Although the vast majority of patients following the recommended interruption strategies achieve low residual anticoagulant levels, trial populations may not fully represent patients with impaired drug clearance or other factors affecting DOAC elimination. In selected patients, those with renal or hepatic dysfunction, heart failure, or uncertainty regarding medication adherence, measurement of anticoagulant activity may identify persistent anticoagulant effect despite reported interruption and provide additional information to guide procedural planning.

## 4 DOAC Measurement Tools

Several laboratory and point-of-care assays are relevant to the evaluation of anticoagulation in patients receiving DOAC therapy. These assays differ substantially in what they measure, turnaround time, availability, and limitations, which are summarized in Table 2.

**Table 2:** Direct oral anticoagulant concentration measurement tools

Test	What it measures	Turnaround time	Advantages	Major limitations
LC-MS/MS Gosselin et al. [7]	Direct plasma DOAC concentration	Usually a send-out test	Gold standard Highly sensitive and specific	Rarely available Labor- and time-intensive
DOAC–calibrated anti-Xa assay Baker et al. [25] Gosselin et al. [7]	Drug-specific plasma concentration	Institution-dependent, sometimes not available 24/7 ∼1 h	Correlates with gold standard	Limited availability Requires drug- specific calibrators Interassay variability Thresholds not outcome-validated
Routine coagulation assays (PT/INR, aPTT, TT) Kitchen et al. [46]	Indirect coagulation effect	Approximately 1 h	Universally available Rapid	Variable sensitivity for DOACs Abnormal results are nonspecific Influenced by many factors
Viscoelastic testing (TEG, ROTEM) Dias et al. [47] Fontana et al. [48] Jenrette et al. [49]	Whole-blood clot formation and clot strength	Approximately 1 h	Provides global assessment of coagulation	Variable sensitivity for DOACs Abnormal results are nonspecific Influenced by many factors
Urine point-of-care testing Harenberg et al. [50] Harenberg et al. [51] Margetić et al. [52] Tan et al. [53]	Presence or absence of DOAC in urine	10-15 min	Rapid, bedside test High sensitivity for DOAC exposure	Qualitative test, does not give a specific concentration Affected by renal function, urine production, hematuria, and hyperbilirubinemia
Whole-blood point-of-care testing Bakhru et al. [54] Bakhru et al. [55] Cuker et al. [56]	Whole-blood clotting time reflecting current anticoagulant activity	10-15 min	Rapid, bedside test No plasma preparation Reflects current anticoagulant activity	Limited clinical validation Cannot distinguish DOAC effect from other causes of coagulopathy Not widely available

aPTT, activated partial thromboplastin time; DOAC, direct oral anticoagulant; INR, international normalized ratio; LC-MS/MS, liquid chromatography with tandem mass spectrometry; PT, prothrombin time; TEG, thromboelastography; TT, thrombin time.

Liquid chromatography with tandem mass spectrometry (LC-MS/MS) is widely considered the gold standard test for measuring DOAC drug concentrations [31]. LC-MS/MS has shown a high degree of sensitivity, specificity, and reproducibility [7]. LC-MS/MS directly measures plasma drug concentration, providing pharmacokinetic information, rather than functional anticoagulant effect. This measurement technique is complex, time- and labor-intensive, and only available in highly specialized laboratories, typically as a send-out test.

Anti-Xa assays measure the overall inhibition of factor Xa, typically calibrated to assess heparin anticoagulation. DOAC-calibrated anti-Xa assays measure DOAC concentration using a specific calibrator for the drug being tested and are comparable with LC-MS/MS [7]. These assays provide similar pharmacokinetic data to LC-MS/MS, and their utility is also limited by availability, time to result, and interassay variability [25]. Though some institutions have DOAC-calibrated anti-Xa assays available, many do not have the assay, or access is limited to specific laboratory hours. When available, clinical turnaround may take up to an hour. Furthermore, their clinical utility is limited by the lack of established therapeutic ranges or thresholds, as discussed above.

Routine coagulation laboratory tests, such as prothrombin time, activated partial thromboplastin time, and thrombin time, are widely available and more rapidly available, unlike LC-MS/MS and DOAC-calibrated anti-Xa assays, but are not reliable for assessing DOAC anticoagulant effect. These assays may provide limited and indirect pharmacodynamic information that does not correlate with DOAC concentration or effect. The sensitivity of these assays to DOACs varies depending on the specific reagents used, leading to significant interlaboratory variability. Furthermore, these values are often impacted by other factors in critically ill patients, such as liver disease, disseminated intravascular coagulation, or coagulation factor deficiencies. In the absence of confounding factors, abnormal values may point toward DOAC exposure as a qualitative measure but cannot quantify levels. But even patients with therapeutic concentrations of apixaban and rivaroxaban may have normal prothrombin time and partial thromboplastin time results [46].

Thromboelastography and rotational thromboelastometry, forms of viscoelastic testing, measure whole blood to provide a comprehensive coagulation assessment, evaluating clot formation and breakdown. These tests are often used to guide resuscitation efforts in high-acuity patients such as those with trauma or major bleeding, suspected coagulopathies, or perioperatively and intraoperatively for major operative interventions, such as transplant and cardiac surgery. Many studies have assessed DOAC impact on clotting time or reaction time in viscoelastic testing, and the evidence is mixed. Prolonged R time was observed compared with control groups for direct Xa inhibitors and correlated with dose for apixaban [47]. Rivaroxaban has shown correlation with clotting time at high levels, but not trough levels [48]. However, in a small retrospective study of 40 patients reporting consistent DOAC therapy and a dose within the past 12 hours, viscoelastic testing was not reliably abnormal [49]. These findings suggest limited sensitivity of viscoelastic testing with regard to DOAC concentrations or anticoagulant effect.

More recently, point-of-care urine-based and whole-blood testing have emerged as methods to assess DOAC exposure and effect. Compared with conventional blood-based laboratory assays, these point-of-care tests offer a significant time advantage, with results within 15 minutes, making them suitable for use in emergent or urgent clinical decision-making [50], [54]. Clinical turnaround times for laboratory assays depend on specimen transport, laboratory workflow, and staffing. In contrast, point-of-care tests can be performed at or near the bedside, minimizing delays related to these logistical factors. The point-of-care urine-based test has demonstrated high sensitivity, specificity, and overall accuracy for the presence of DOAC [51]. Notably, one cohort study including 124 DOAC-treated individuals reported 100% sensitivity and negative predictive value of the DOASENSE DOAC Dipstick point-of-care test for detecting DOAC plasma concentrations greater than 30 ng/mL, determined by DOAC-calibrated anti-Xa serum testing. Of note, samples in this study were trough values, obtained just prior to the expected morning dose [52]. In another prospective cohort study of 265 patients treated with DOACs presenting to the emergency department, point-of-care urine-based testing was compared with the gold standard for determining DOAC serum concentration, LC-MS/MS. Data similarly showed high sensitivity, 100% for dabigatran and 97% for the apixaban and rivaroxaban group, of the point-of-care urine test for detecting DOAC concentrations greater than 30 ng/mL. Importantly, the negative predictive value for the apixaban and rivaroxaban group was 90% (95% CI 79.3-96.9) for detecting DOAC levels greater than 30 ng/mL, indicating that 10% of patients with a negative point-of-care urine test may have a DOAC serum level greater than 30 ng/mL [57]. This is an important clinical consideration if this test were to be used to rule out the DOAC anticoagulant effect.

This point-of-care urine test has been proposed for use in acute stroke care, where rapid determination of anticoagulant presence may enable timely use of thrombolytic therapy in patients who would otherwise be excluded due to reported or suspected DOAC use [53]. It is important to note that the pharmacokinetics of urinary DOAC excretion are not fully understood and can be influenced by renal function and the timing of the last dose. Severe renal impairment may lead to reduced drug excretion in the urine, potentially resulting in false-negative results. Additionally, test results may reflect anticoagulation presence from hours prior, at the time the urine was produced, rather than the time of testing [51]. Lastly, hematuria and hyperbilirubinemia may alter the color and thus affect the result of this urine test, although control strips attempt to limit this impact.

A whole-blood point-of-care test, the Perosphere ClotCheck, is a pharmacodynamic assay that assesses individual patient DOAC effect by measuring ClotCheck Clotting Time [58]. It is CE-marked and available in Europe. This assay requires no reagents and uses ∼14 μL of whole blood, not plasma. *In vitro* studies using healthy whole blood spiked with varying concentrations of DOAC demonstrated a strong linear correlation between clotting time and DOAC concentration, with high sensitivity even at low drug levels [54]. In a clinical validation study, which included patients on rivaroxaban or apixaban along with drug-naive healthy individuals, the ClotCheck demonstrated high sensitivity at trough and peak DOAC concentrations and a strong linear correlation between clotting time and concentration as measured by anti-Xa assay. It distinguished both rivaroxaban and apixaban anticoagulation from normal with a high degree of confidence using a 75 ng/mL DOAC threshold as well as using the lower 30 ng/mL DOAC threshold with similarly high confidence [55]. Other whole-blood point-of-care testing devices in development include iLine microDOAC, a semiquantitative analysis of DOAC concentration [56].

Whole-blood point-of-care testing offers pharmacodynamic data and, unlike urine-based testing, reflects current anticoagulant activity and is not dependent on renal excretion. Of note, it does not distinguish between the cause of impaired coagulation and may be abnormal in acquired or congenital coagulopathies. Clinical data are evolving, and further studies are underway to determine accuracy and reliability in clinical use in patients treated with DOACs.

## 5 Future Directions and Newer Therapies

As evidence supporting DOAC measurements continues to grow, future efforts should focus on optimizing their implementation in clinical practice. Prospective studies are needed to determine whether incorporation of DOAC measurement into clinical decision-making improves patient-centered outcomes, including bleeding complications, thrombotic events, and time to intervention. Additional research is also needed to better define clinically meaningful drug concentration thresholds across diverse clinical scenarios.

Development of institutional decision-support pathways, standardized order sets, and anticoagulation stewardship programs may facilitate assay selection, timely specimen collection and processing, result interpretation, and integration into clinical decision-making. Future implementation strategies should also emphasize multidisciplinary collaboration and transition-of-care planning, including communication regarding anticoagulation resumption following abnormal results, bleeding events, or interventions such as thrombolysis or reversal.

Emerging anticoagulants, including factor XI inhibitors, may further reshape anticoagulation management and alter future approaches to laboratory testing [59], [60]; however, additional investigation is needed to determine appropriate laboratory testing and management strategies for these agents in patients with major bleeding or requiring urgent intervention.

## 6 Conclusion

DOACs have transformed anticoagulation management by providing effective and safe therapy without the need for routine laboratory monitoring. The population of patients receiving DOACs and other anticoagulation therapies is projected to grow with the aging population, the introduction of generics, and anti-XIa inhibitors with new indications. While DOACs’ fixed dosing simplifies chronic management, the lack of readily available means to measure DOAC anticoagulant effect in various clinical settings can pose a challenge. AIS, major bleeding, and perioperative care are scenarios in which uncertainty regarding the DOAC effect can prevent, delay, or complicate management; likewise, visibility into a patient’s coagulation status may aid in optimal clinical decision-making, answering key questions as shown in Table 3. Routine coagulation laboratory tests and viscoelastic testing do not reliably assess the DOAC anticoagulant effect. Laboratory methods that measure DOAC concentration, including LC-MS/MS and DOAC-calibrated anti-Xa assays, are constrained by limited availability, prolonged turnaround times, and, most notably, lack of outcome-validated thresholds. Consequently, DOAC concentrations, when available, must be interpreted in the context of this limitation. As DOAC use continues to rise, clinicians increasingly encounter situations in which rapid assessment of anticoagulant effect could meaningfully support improved clinical decision-making. Emerging point-of-care testing modalities offer a tool to address these challenges by providing timely and more DOAC-specific information for clinicians in these scenarios. Important limitations remain, and further studies are needed to determine how rapid assessment of the DOAC anticoagulant effect can be safely and effectively integrated into clinical care.

**Table 3:** Role of direct oral anticoagulant concentration measurement in key clinical scenarios.

Scenario	Question to answer	When measurement may be most useful	Potential impact on management
Acute ischemic stroke	*Is the degree of DOAC anticoagulation low enough to administer IVT or to perform intracranial interventions?*	Suspected DOAC use Incomplete medication history (due to stroke symptoms or underlying cognitive deficits) Unknown time of last dose	Absent or very low DOAC levels may allow advanced therapy to proceed
Major bleeding	*Is the degree of DOAC anticoagulation low enough to avoid reversal?* *Is the degree high enough to warrant reversal?*	Life-threatening or uncontrolled bleeding Suspected DOAC use Incomplete medication history (due to severe illness or underlying cognitive deficits)	Absent or very low DOAC levels may preclude need for reversal
Emergent/urgent surgery	*Is the degree of DOAC anticoagulation low enough to avoid reversal?* *Is the degree high enough to warrant reversal?*	Immediate intervention required Suspected DOAC use Incomplete medication history (due to severe illness or underlying cognitive deficits)	Absent or very low DOAC levels may preclude need for reversal Knowledge of level can help in surgical planning
Elective surgery	*Is the degree of DOAC anticoagulation low enough to proceed with routine or elective intervention?*	Uncertain adherence to interruption	Inform whether to proceed with or postpone planned interventions

DOAC, direct oral anticoagulant; IVT, intravenous thrombolysis.
